# Generalising from conventional pipelines using deep learning in high-throughput screening workflows

**DOI:** 10.1038/s41598-022-15623-7

**Published:** 2022-07-06

**Authors:** Beatriz Garcia Santa Cruz, Jan Slter, Gemma Gomez-Giro, Claudia Saraiva, Sonia Sabate-Soler, Jennifer Modamio, Kyriaki Barmpa, Jens Christian Schwamborn, Frank Hertel, Javier Jarazo, Andreas Husch

**Affiliations:** 1 National Department of Neurosurgery, Centre Hospitalier de Luxembourg, 4, Rue Ernest Barble, 1210 Luxembourg (City), Luxembourg; 2 Interventional Neuroscience Group, Luxembourg Center for Systems Biomedicine, University of Luxembourg, 6, Avenue du Swing, 4367 Belvaux, Luxembourg; 3 Developmental and Cellular Biology, Luxembourg Center for Systems Biomedicine, University of Luxembourg, 6, Avenue du Swing, 4367 Belvaux, Luxembourg; 4 OrganoTherapeutics SARL, 6A, avenue des Hauts-Fourneaux, 4365 Esch-sur-Alzette, Luxembourg; 5 Systems Control Group, Luxembourg Centere for Systems Biomedicine, University of Luxembourg, 6, Avenue du Swing, 4367 Belvaux, Luxembourg

**Keywords:** Computational biology and bioinformatics, Systems biology

## Abstract

The study of complex diseases relies on large amounts of data to build models toward precision medicine. Such data acquisition is feasible in the context of high-throughput screening, in which the quality of the results relies on the accuracy of the image analysis. Although state-of-the-art solutions for image segmentation employ deep learning approaches, the high cost of manually generating ground truth labels for model training hampers the day-to-day application in experimental laboratories. Alternatively, traditional computer vision-based solutions do not need expensive labels for their implementation. Our work combines both approaches by training a deep learning network using weak training labels automatically generated with conventional computer vision methods. Our network surpasses the conventional segmentation quality by generalising beyond noisy labels, providing a 25% increase of mean intersection over union, and simultaneously reducing the development and inference times. Our solution was embedded into an easy-to-use graphical user interface that allows researchers to assess the predictions and correct potential inaccuracies with minimal human input. To demonstrate the feasibility of training a deep learning solution on a large dataset of noisy labels automatically generated by a conventional pipeline, we compared our solution against the common approach of training a model from a small manually curated dataset by several experts. Our work suggests that humans perform better in context interpretation, such as error assessment, while computers outperform in pixel-by-pixel fine segmentation. Such pipelines are illustrated with a case study on image segmentation for autophagy events. This work aims for better translation of new technologies to real-world settings in microscopy-image analysis.

## Introduction

High-throughput High-content screening is a powerful tool in systems biology, thanks to its capacity to quantitatively measure the dynamical behaviour of biological processes using fluorescence microscopy^1^. For this task, image analysis is a crucial step that requires handling hundreds of images generated every day. Therefore its automatic processing has become a paramount objective. In the literature, most Deep Learning (DL)-oriented academic papers tackling image analysis frequently employ highly curated benchmarking datasets. Such works focus solely on increasing the accuracy of the algorithms. Although this goal has been crucial for the fast development of the methods during the last years, working with real-world datasets brings new challenges. These challenges include label quality, such as noisy label data, or incorrect segmentation^2^; additionally, manually curated labels are not only time-consuming but also a complex task in biomedical datasets^3^. Finally, the developed solutions are generally difficult to use. All these issues hamper the real use of DL-based solutions in everyday laboratories.

Computer Vision (CV) techniques for digital image processing have undergone a remarkable evolution since their first developments in the 60s^4^. One of the most relevant advances includes the employment of Artificial Intelligence (AI) methods, which became especially important after the first big success of a Convolutional Neural Network (CNN) with ImageNet in 2012^5^. The main difference between traditional CV and AI-based solutions is the paradigm behind it. On the one side, traditional CV is descriptive, requiring a definition of a comprehensive mathematical model to describe the phenomenon that we wish to model. In image analysis, this entails employing different filters and parameters, i.e. a hand-crafted feature definition approach. On the other side, predictive analysis builds upon the automatic discovery of the rules that underlie the studied phenomena, such as optimising operations to minimise the error between the actual and the predicted outcome^6^.

HTS has been traditionally addressed using pipelines based on conventional image processing (from here on referred to as CIP) techniques such as thresholding, morphological operators, contour based^7^ or graph-cut^8^ algorithms. However, as mentioned before such approaches require expertise, time and handcraft to develop *ad-hoc* pipelines that need to be adjusted to each case, hindering generalisation. Alternatively, as in many other fields in the last years, the use of Machine learning (ML) techniques has become very popular for image analysis. In particular, deep learning-based solutions^9^, such as CNN now dominate the field due to the superiority of their results^10^. Considering that CNN-based solutions outperform traditional algorithms thanks to the increasing computer power and dataset availability^6^, it may seem that traditional CV techniques are obsolete. Nevertheless, the latter approaches do not require complex and costly labels for its development compared to CNN-based solutions. Moreover, most CNN approaches are based on images manually analysed by humans (including event segmentation or even semantic segmentation), which is not only very time consuming but due to the nature of the biological data, good quality segmentation at the pixel level is hard to obtain^11,12^. This renders CIP algorithms still relevant, since images are automatically generated (e.g. using an HTS system), can be processed with techniques that require simple thresholding and basic corrections yielding an acceptable quality of segmentation^13^.

Even though Supervised ML requires large amounts of data, several approaches such as transfer learning techniques^14^ or data augmentation techniques, ranging from geometric transformations or colour space augmentations to generative adversarial networks^15^ can overcome this limitation. These techniques can be paired with other strategies such as the automatic generation of labels, which may lead to noisy labels, also known as CIP based DL (CDL) approaches. Training with noisy labels is a common problem in the supervised ML community due to the high cost of properly curated datasets. This is especially relevant when the labelling task requires domain-specific knowledge such as biological or medical data^2^.

Although a common scenario, working with noisy label data presents several difficulties during both the training and the evaluation of models. Previous works have shown that CNN can generalise even when there are noisy data in the training datasets, overcoming such inaccuracies^16^. Furthermore, the choice of the cost function is an essential step during the solution design. This choice depends on the problem and dataset characteristics. For instance, in class imbalance scenarios, the employment of class sensitive cost functions such as dice-coefficient is recommended^17^. Additionally, dice-coefficient is considered a robust cost function in scenarios with noisy datasets^18^.

In recent years, different strategies to approach the annotation problem have been developed. Here we highlight active learning, continuous learning, curriculum learning and knowledge distillation. Active learning refers to the concept of reducing the annotation effort needed to train a model while still maintaining good performance by actively selecting the most informative or representative samples. Multiple approaches have been proposed to find the most representative examples, such as selecting the most influential items^19^.Active learning strategies have been successfully applied on different problems including multiple instance labelling for classification tasks^20^, regression tasks^21^ and in DL solutions^22^. Other approaches include continuous learning. This paradigm refers to the model’s ability to continually learn from stream data, as a way to update the model during the production phases keeping its relevance and performance. Continuous learning is especially relevant in scenarios where data is constantly changing^23^. In the biomedical image analysis context, this approach has been proposed in ML-assisted diagnostic tools for hospitals where the algorithm performance degrades over time. The root cause in many cases is the expected changes in local data, such as data acquisition pipelines and population shifts, reducing the risk of bias and errors over time.^24^. In the same line we can find the concept of curriculum learning. Humans and other animals are able to learn complex ideas more efficiently when gradually learned from more basic to the more complex ones^25^. Recent works in this area have been successfully implemented in skin lesion segmentation^26^ and histopathology image classification^27^. Finally, Knowledge distillation refers to the concept of transferring the information learned from one model (generally larger) to another (usually smaller) also known as ’Student teacher’^28^. Although this concept was initially developed focusing on optimising the resources used from a smaller network, several works suggest that this can also be employed to improve the generalisation and robustness in the context of noisy label data^28–30^.

The Machine Learning (ML) community aims to develop *automatic* ML with the ultimate goal of bringing humans-out-of-the-loop, such as autonomous vehicles. However, domain expertise can be seen as an external agent on the interactive ML, including human-in-the-loop, allowing humans to obtain a synergistic combination of methodologies^31^. This could help to avoid the uncertainty and incompleteness, including noisy data, seen in biomedical datasets.

In this paper, we propose a human-in-the-loop pipeline including both traditional and AI-based computer vision methods. This approach was inspired by the capacity of deep neural networks to overcome noisy labels, as extensively explained in^32^. For this purpose, we designed an alternative solution to CIP involving DL methods. We tackle two common scenarios which differ on the nature of the training data and its quantity. The first approach employs a large training set of automatically generated labels, CIP based DL, from here on referred to as CDL. In contrast, the second approach relies on a small manually curated dataset, Manually curated based DL, thereby referred to as MDL. We illustrate the use of these pipelines in a case study for the segmentation of HTS microscopy images. This dataset includes imaging samples of the autophagy pathway using the Rosella pH-sensitive biosensor using human iPS cells.

### Case study

Autophagy is an evolutionary conserved catabolic process that mediates the degradation of dysfunctional or useless organelles in eukaryotic cells. Autophagy has as well an essential role in maintaining homeostasis in stress conditions^33^. Accurate frameworks to measure autophagy are still an open research question. However, “autophagy flux”, in which the different autophagy phases can be measured, has been established as one of the best approaches to study autophagy in pathological conditions. Our dataset uses the Rosella pH-sensitive biosensor^34^ that allows for the identification of the different autophagy phases^13^. The gold standard in segmentation is the manual creation of label masks by experts. Notwithstanding, such an approach requires a large amount of work from highly trained researchers. An existing solution to the segmentation problem of autophagy events is based on CIP techniques specifically optimised for fluorescent microscopy analysis of cells with the Rosella biosensor to report autophagy events as suggested in^13^. In this study, we demonstrate how to effectively leverage this approach developing two alternative pipelines (CDL and MDL) to improve the final segmentation quality.

## Methods

This section is structured into three main parts. First, a general overview, dataset generation and CNN architecture are described. The next section focuses on the CDL (CIP based DL) approach development, including the evaluation of the expected generation capacity of CNN for overcoming errors. The last part covers the MDL (Manual curated based DL) approach implementation and holistic evaluation of the three strategies: CIP, CDL and MDL.

### Pipeline setup

**Figure 1:**
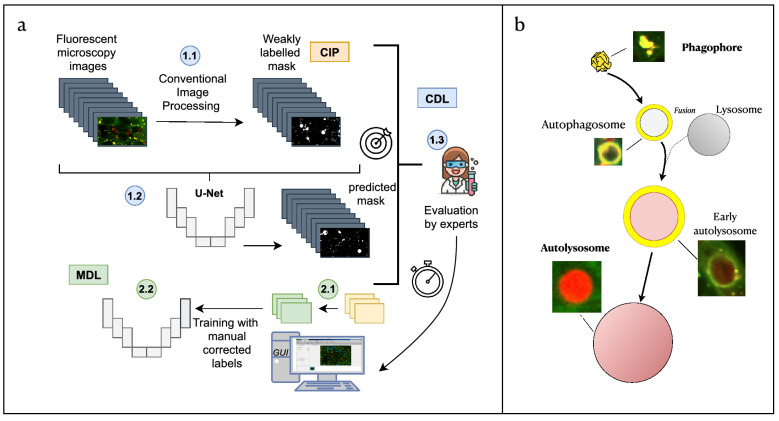
(**a**) General workflow of the proposed pipeline, part 1: (In blue, CIP-based DL: CDL). [1.1] First a weakly labelled dataset is created using conventional imaging processing (CIP). [1.2] After that, a U-net like architecture is trained and [1.3] the accuracy of the evaluated. Including an integration of the trained network in an intuitive tool for biologists that allows easy correction. Proposed pipeline, part 2: (In green, for Manually based DL: MDL). [2.1] Manual corrected masks are easily generated using the GUI, which is employed to train a U-net from scratch [2.2]. (**b**) The biological process of autophagy and its detection with Rosella biosensor. The four main phases are: The initial state - Phagophore, intermediate states - Autophagosome and early autolysosome, and final state - Autolysosome. The fusion with the lysosome during the autophagy process yields a pH decrease which induces a change of colour in the fluorescent microscopy image.

The different pipelines are represented in Fig. 1a. The top part of the panel corresponds to the CDL method while the bottom part to the MDL. The CDL approach is divided in three steps: (1) Labels are automatically generated using CIP techniques; (2) These masks are employed to conduct a supervised train using a CNN. As mentioned before, a good generalisation that overcomes the systematic incorrectness of the weakly labelled data is expected. (3) Evaluation of the generalisation can be done by using alternatives metrics explained in the evaluation section. This trained network is introduced in an easy-to-use Graphical User Interface (GUI) allowing the user to predict new images using a considerable less amount of time and computational resources than CIP analysis, and being more precise thanks to its generalisation over the errors. Using the GUI tool, the experts can also correct the potential inaccuracies of the images. The reduced time needed to correct the current prediction in comparison with the semantic segmentation from scratch is also a considerable benefit. The two-steps MDL pipeline is organized in two steps: (1) using the previously developed GUI, masks are predicted and manually corrected, (2) Using this small manually corrected dataset a CNN is trained (1a bottom part). Finally, the tree methods, CIP, CDL and MDL were evaluated.

### hiPSC generation and imaging acquisition

Generation of the hiPSCs lines, imaging obtainment was performed as previously described^13^. The human iPSCs line used in this study is A13777 obtained from Gibco. Briefly, the hiPSCs gene-edited with the Rosella construct into the AAVS1 safe harbour were cultured in Essential-8 media (Thermo Fisher cat no. A1517001) in CellCarrier Ultra plates (Perkin Elmer, 6055300). Confocal images were obtained with an Opera QEHS spinning disk microscope (Perkin Elmer) under a 60x water immersion objective ($NA\cdot =\cdot 1.2$). DsRed and pHluorin images were acquired simultaneously using two cameras and binning 2.

### CIP pipeline

In this paper, we refer as CIP to a previously developed image analysis workflow described in detail in^13^. It is “conventional” in that it employs the traditional techniques in computer vision to conduct semantic segmentation. Hence, it is not a model trained using machine learning but a processing pipeline manually engineered by a human expert. Examples of techniques used as building blocks to create such pipelines are: image deconvolution^35^, thresholding^36^ Gaussian filtering^37^, Top-hat filtering^38^, watershed transformation^39^, difference of Gaussians^40^, Butterworth filter^41^ or high pass filter^42^. The CIP pipeline was implemented in MATLAB and was specifically designed and fine-tuned for the case study of Autophagy. As a result, the segmented vesicles were classified into 1 of 4 categories (phagophores, autophagosomes, early autolysosomes, and late autolysosomes) based on the obtained masks and the pixel intensity. The autophagy process with the aforementioned steps and its aspect on the microscopical images is depicted in Fig. 1b. In this paper, we focus on the two main categories (Phagophares and Late autolysosomes).

### Network design

Our solution employs a multi-class semantic segmentation network^43^ based on the U-net, which is one of the most common architectures in the state of art for semantic segmentation to process biomedical images^44,45^. The U-net network architecture is a symmetric encoder-decoder architecture with skip connections. In the encoder part, the image features are extracted through a combination of convolution and pooling operations. Then, the decoder part builds the segmentation output. By using skip connections the input image resolution is preserved for the output label masks, ensuring detail conservation. The employed network contains a total of 3 max-pooling and 3 skips connections. To facilitate the learning on the strong class imbalance scenario, a specific class-sensitive loss function was selected^46^. The generalised dice coefficient^47^ was included as a cost function for the final pixel classification layer. The employment of the U-net architecture is consistent with recent results in medical imaging, where classical U-Net architectures were found to have excellent generalisation performance across different segmentation tasks^48^. A graphical representation of the employed network is depicted in Supplementary Material 1.A.

### Part A: Measuring the DL generalisation robustness with noisy label data for semantic segmentation

The first part of the paper presents the development of the CIP-based DL approach: CDL. We start from a previously existing pipeline based on CIP that have some inaccuracies and train a DL model, expecting benefits for the generalisation capacities of DL. To measure this, we designed three different strategies to quantified the generalisation capacity described in the evaluation section.

#### Data pre-procesing

Since the exploratory data analysis showed a strong class imbalance between the background and the classes of interest (frequency was $0.95/0.023/<0.001/0.001/0.024$ for background, phagophore, autophagosome, early autolysosome and autolysosome, respectively), in this paper, we focus on the three most frequent classes, phagophore, autolysosome and background. The discarded classes, the autophagosome and early autolysosome stages, have a extremely low-frequency^13^ and thus very limited available training data for these instances. The dataset used for training was formed by 4000 HTS images of 680 × 512 × 2. The two microscopy channels were encoded as the red and the green channel in RGB data. Normalisation^49^ and data augmentation techniques (random reflection and rotation)^50^ were employed to increase the dataset diversity, reducing the risk of over fitting during the model training.

#### Training

The network was trained in MATLAB, including MATLAB Deep Learning, Computer Vision and Parallel Computing toolbox. We decided to use MATLAB instead of DL community more widespread frameworks as we aimed from the beginning to provide a tool that works on real data and that can be utilised to help biologists in their daily routine by integrating the CNN based segmentation in graphical MATLAB tools used for image analysis. The dataset was split 0.85/0.1/0.05 for training, validation and test respectively. Stochastic gradient descendent with momentum of 0.9^51^ was used as optimiser with L2 regularisation^52^ of 0.001 and an initial learn rate of 0.002 with a learn rate drop factor of 0.8 every 3 epochs. The mini-batch size was reduced to 4 due to the large input image size and limited GPU memory. The network was trained for 15 epochs from scratch.

#### User-friendly GUI

Next, the CNN was integrated into a user-friendly tool using the MATLAB Image Label App^53^. This integration allows easy handling for the potential users, biological researchers that often do not have experience with programming. By integrating the CNN as a segmentation algorithm into the image label app the tool does not only allow for an easy prediction of the mask using the CNN solution but also an intuitive way to correct the errors of the mask using manual segmentation tools. The image label app is usually a known environment for potential users providing the typical tools for manual refinement (like brush tools, lasso tools). An example of the GUI is shown in Supplementary Material 1.B.

#### Manual correction

Generation of manual labels from scratch is a hard and expensive task. Instead, the images were firstly predicted with our trained network and then manually corrected using the tools available in the GUI. The manually corrected dataset of 306 quarters was done by four biologists with experience in cell microscopy.

#### Evaluation

Evaluation of the segmentation task on noisy label conditions can be challenging because of the lack of an accurate gold standard. The present evaluation aim is twofold. On the one hand, attempts to quantify the generalisation capacity of deep neural networks for microscopy using automatically generated weakly labels. On the other hand, to assess the effectiveness of the proposed framework, accounting for its application to analogous situations. The capacity of generalisation is evaluated using three complementary but independent strategies: (1) a qualitative analysis using blind expert ranking. (2) a quantitative analysis using Bounding Boxes (BB) as a surrogate metric.(3) a qualitative analysis using the overlapping segmentation using dice-coefficient employing manually corrected samples. Finally, our proposed solution was analysed from a time and computational resources perspective in the performance comparison section.

##### Evaluation: qualitative analysis by expert rating

Double-blind randomised tests were conducted by four experts in cell biology with experience in this type of image. The expert quantification was performed in RGB microscopy images with adjacent plots of two label masks of that image as well as an overlay of the image with the masks. The masks were either produced by CIP or CDL and the experts were blinded about the randomised order of the segmentation. They scored each labelling from 1 to (worst quality) to 10 (best quality). A total of 40 images/80 segmentation were split into two subsets of 20 images each. Each subset was presented to two experts, thus each expert rated 40 segmentation masks from 20 images; An example of the test employed is depicted in Supplementary Material 1.C. To assess the significant difference, the Kolmogorov-Smirnov test^54^ was employed.

##### Evaluation: quantitative analysis: accuracy detection as a surrogate metric

Detection and segmentation are considered different tasks in traditional computer vision literature, however, segmentation implies detection^55^. Hence, detection of the events can be employed to quantitatively assess segmentation accuracy as a surrogate metric. Among the benefits of this approach, the creation of a manual detection ground truth for analysis is much less time-consuming and more tolerant of the potential errors during the creation of the dataset. A sample of 100 HTS microscopy images was reviewed by two separate groups of experts and labelled by surrounding each event with a BB, hence a total of four experts. Then, the BBs were automatically extracted from both the CIP mask and the CDL mask. Next, the expert manually labelled BBs were compared against CIP and CDL respectively. Intersection-over-union of the BBs was employed to measure the same detection, only considering intersections equal or bigger than 0.1 of overlapping in order to remove the effect of random overlapping. To quantify which method performs better, two type of analysis were performed. First, the number of BBs that do not overlap, in other words the analysis of the two type of errors; Second, the number of elements that overlap. For the first one, the ratio of events detected by the methods (CIP or CDL) but not for the humans (false positive - Type I of error) and the events detected by the humans but not by the evaluated method (false negative - type two of error). This was analysed considering the ratio of events for each of the samples labelled by each expert. Lower values in the ratio of events for the method that performs better detection are expected. For the second one, the mean and standard deviation of the total number of events detected was employed.

The overlap was analysed using two different ranges, lower overlap (from 0.1 to 0.49) and higher overlap (from 0.5 to 1).

##### Evaluation: quantitative analysis—dice coefficient comparison

While the human rating of the segmentation and the detection analysis are good approximations of segmentation’s quality assessment, the best comparison is segmentation labels as gold standards. Nevertheless, as mentioned before its generation is time-consuming. The manually corrected dataset was employed as the gold standard to compare, the CIP and the CDL generated mask using the Sørensen-Dice coefficient.^56^.The following metrics are employed to compare the semantic segmentation, including accuracy (Eq. 1), precision (Eq. 2), recall (Eq. 3), specificity (Eq. 4), intersection over union (IoU) (Eq. 5) and the boundary F1 score (BFS)^57^(Eq. 6), formulas in table 1. Since IoU is employed, Dice coefficient was not included since its correlation with the Sørensen-Dice coefficient will not provide additional information to compare the approaches^58^. Additionally, the metrics are aggregated in three different ways: ’Global’ being the ratio of correctly classified pixels, regardless of class, to the total number of pixels; ’Mean’, as the average score of all classes in all images; and ’Weighted’ by the number of pixels in each class. Considering that our dataset has a strong class imbalance, the ’Mean’ aggregation and the disaggregated (i.e. per class) convey the fairest comparison.

**Table 1:** Employed Metrics.

$$Accuracy = TP/(TP + TN)$$	(Eq. 1)
$$Precision = TP/(TP + FP)$$	(Eq. 2)
$$Recall = TP/(TP + FN)$$	(Eq. 3)
$$Specificity = TN/(TN+FP)$$	(Eq. 4)
$$IoU = TP/ (TP + FP + FN)$$	(Eq. 5)
$$BFS = 2 * Precision * Recall / (Recall + Precision)$$	(Eq. 6)

### Part B: Semantic segmentation performance evaluation of CIP, CDL, and MDL approaches

The second part of this work focused on the development of the MDL strategy, a DL-based solution trained with the manually corrected dataset as previously described. Next, using the test set the accuracy of the previously developed network, CDL and the CIP method were compared with the MDL approach to assess its accuracy.

#### DL approach and training

The two-step workflow is represented as MDL in Fig. 1a bottom part. Firstly, the manually corrected masks are easily generated using the GUI. Then (2) this dataset is employed to train a U-net from scratch similarly to the network training in the CDL approach. The DL network was trained from scratch using the manually curated data set during 15 epochs. The dataset was divided using 90% (80% for training and 10% for validation) and kept the 10% for the posterior testing. A stochastic gradient descendent with 0.9 with momentum was employed. As optimiser with L2 regularisation of 0.001 and an initial learning rate of 0.001 with a learn rate drop factor of 0.8 every 3 epochs. As before, the mini-batch size was reduced to 4 due to the large input image size and limited GPU memory.

#### Evaluation

In this work, we do not only aim to develop an efficient solution in terms of accuracy, resources economy, and usability but also to compare strategies commonly employed when planning to include AI into the automatic image analysis workflow. In this section, we compared the accuracy of the three methods, CIP, CDL, and MDL. To do so, a subset of the manually curated dataset (10%) was employed. Each approach (CIP, CDL and DL) was quantitatively compared using dice coefficient.

### Performance comparison

Although accuracy is essential, time and resource efficiency are also fundamental for a holistic evaluation of the different approaches. Time was evaluated considering three stages: The time for dataset preparation, time for solution’s development, and inference’s time. Resources estimation include human and computational resources. For the first one, we consider resources invested on the critical evaluation, BBs generation, manual correction, and manual curation (from scratch). For the second one, the time needed to program the CIP pipeline and the two DL approaches. Finally, the time need and resources needed such as special GPU resources or conventional resources for the inference.

## Results

This section is structured into two main parts: (1) the generalisation capabilities of a DL network trained on noisy label data generated by a CIP pipeline were evaluated using three independent metrics. (2) the comparison of the three methods: traditional CV methods (CIP), the previously introduced DL trained on noisy labels generated using an existing CIP pipeline (CDL) and DL trained on a small manual curated dataset (MDL).

### Part A: Measuring the DL generalisation robustness with noisy label data for semantic segmentation

**Figure 2:**
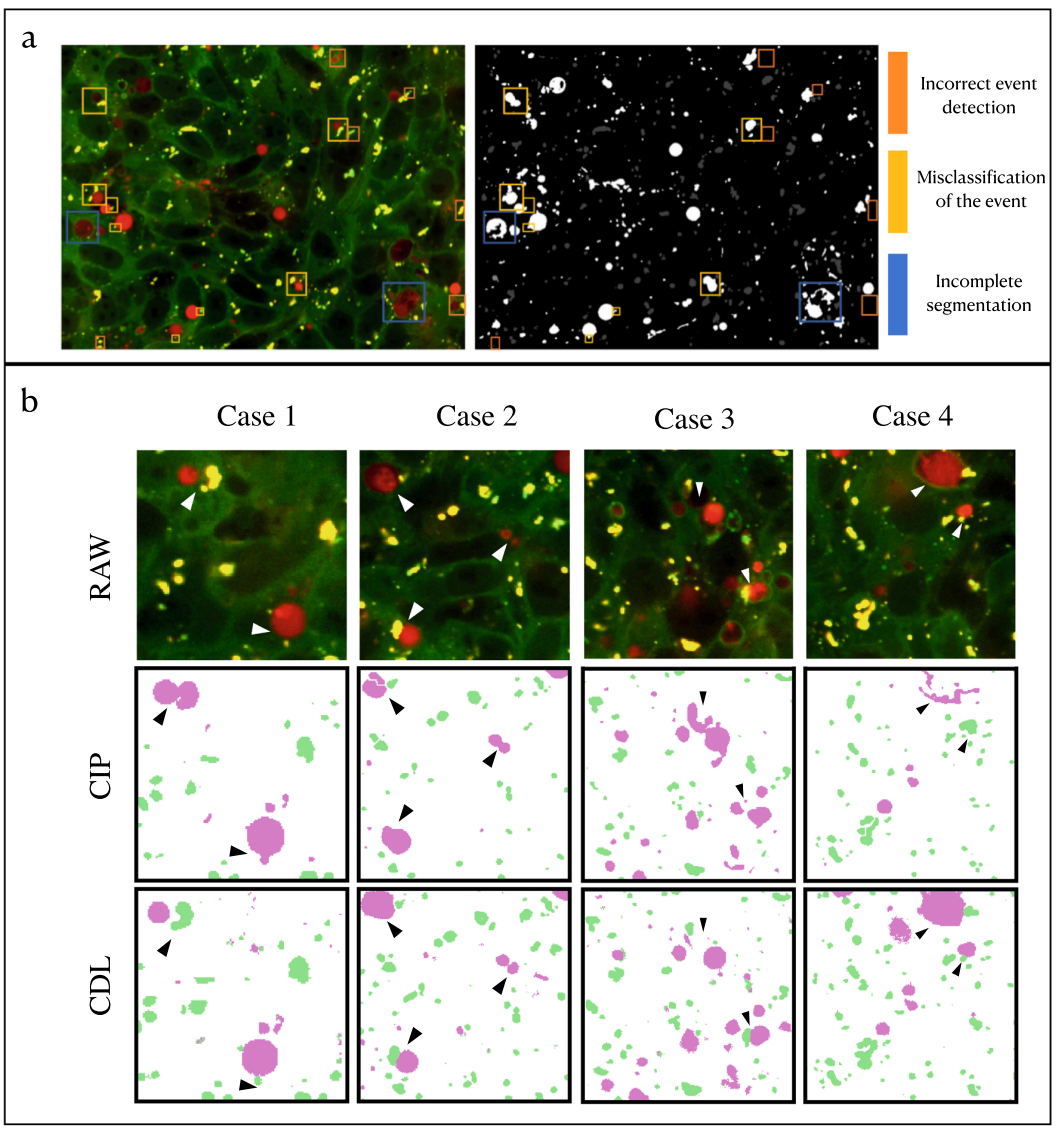
Qualitative analysis of the generalisation capability of CDL vs CIP. (**a**) On the left, a sample raw image from the dataset of fluorescent microscopy images (microscopy channels one and two are displayed as red and green channels of the RGB image). On the right, the corresponding (weak) mask is produced with the conventional image processing (CIP) pipeline. The different types of errors are shown in the legend. (**b**) Examples comparing CIP and CNN based segmentation (CDL) on previously unseen data. Top row: part of original HTS image, middle row: CIP Segmentation, bottom row: CDL segmentation. Errors of the segmentation are highlighted by black arrows. Note the improvements the CNN learned over the CIP which was used to generate training data.

In the CDL approach, a previously existing pipeline described in Arias et al.^13^ was employed as starting point to train the CDL network. This CIP pipeline consisted of a series of heuristically determined filters and operations applied to the images such as flatfield corrections, image deconvolution, difference of Gaussians and thresholding. While this pipeline yields acceptable results, several inaccuracies were found in the resulting segmentation labels when applying them to the data. Such inaccuracies can be classified into three main categories, sorted from the most severe to the most subtle: Missing detection of the event, misclassification of the event and incomplete segmentation. The different types of errors are represented in Fig. 2a. Visual comparison between the labels generated with CIP (used as a noisy label) and the trained CNN (CDL) is depicted in Fig. 2b. In this Figure, we can visually observe that CDL segmentation is out-performing CIP segmentation masks. Afterwards, three different strategies were employed to objectively measure the CNN generalization capacity over the errors presented in the training masks.

#### Qualitative analysis: expert rating

**Figure 3:**
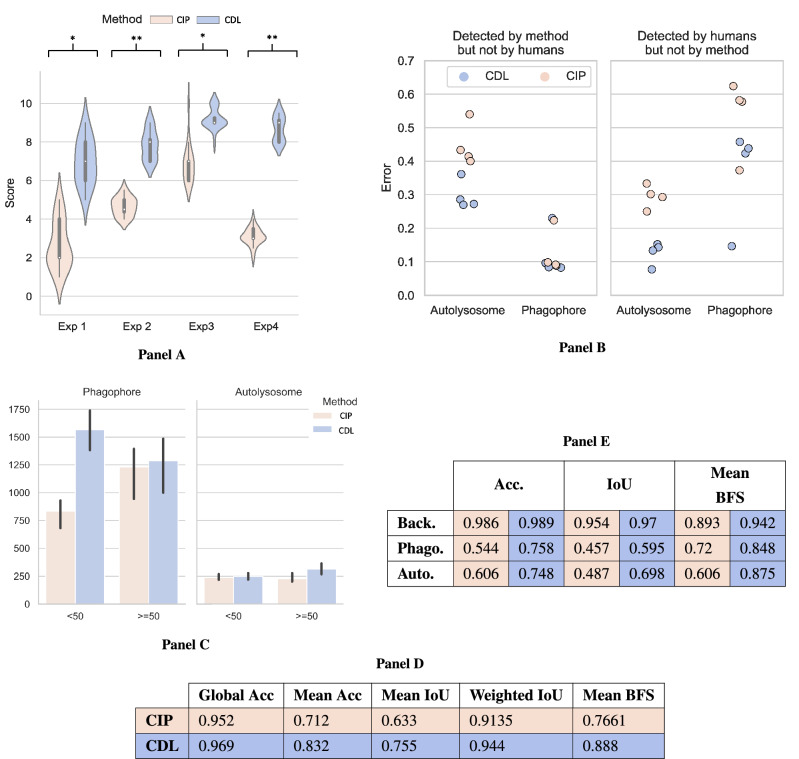
Measuring Deep learning (DL) generalisation robustness with noisy label data for semantic segmentation. Result of the three complementary methods assessing the Convolutional Neural Network (CNN) capacity of overcoming segmentation errors. Comparison of CNN deep learning (CDL) prediction and Conventional image processing (CIP, Method for label generation). (**A**) Results of the qualitative analysis: Expert rating. Violin plots represent the distribution of the score given to each method in the double-blinded test.The CDL method scored significantly higher in all the expert. (**B**,**C**) Results of the quantitative analysis: Detection as a surrogate metric. (**B**) Scatter plots representing the misclassification ratio of a method (CIP or CDL) compared to human annotations. Each point represents the result of a given expert for a given class and method. Therefore, there are 4 points, one for each expert, per class and method. The highest the error ratio, the worse the method performance. (**C**) Results of the quantitative analysis: Detection as a surrogate metric. Plots represent the average number of Bounding Box intersections between the manual reference generated by the four experts and the evaluated method. Error bars represent standard deviation. Overlapping levels split as follows: On the left, lower overlap (between 0.1 and 0.49) and on the right, higher overlap (0.5 to 1). For both classes (Phagophore and Autolysosome) and both overlapping ranges, the number of detected events is higher in the CDL segmentation than in the CIP one. (**D**,**E**) Results of the quantitative analysis: Detection as a surrogate metric CIP results are presented in light orange, CDL results in dark blue. (**D**) Aggregated metrics. (**E**) Metrics broken-down per class. The CDL method showed a better performance in line with the results obtained with the previous methods.

The qualitative/semi-quantitative analysis by expert yielded a mean quality rating of 4.3 for CIP and 8.3 for CDL (1–10, where ten is best). It should be noted that the average rating per expert was significantly varying, giving a notion of different ”critical attitude” of the different experts. The detailed results are shown in Fig. 3A. The Kolmogorov-Smirnov test determined that the score associated per segmentation method within the same experts was significantly different in all the experts, with a p-value ranging from 1e−8 to 1e−11. There were no significant differences between datasets 1 and 2.

#### Quantitative analysis: detection accuracy as a surrogate metric

The quantitative analysis of detection overlap as a surrogate metric is presented in Fig. 3B, represent the ratio of BBs that do not overlap. Each point represents the total number of events detected by BBs in the 50 images that were labelled by the experts in cell biology. On the left, detected by the method (CDL or CIP) but not by humans (false positive). For the BBs that detected autolysosome, the mean ratio of events is 0.44 (0.06 std.) for CIP and 0.29 (0.04 std.) for CIP, hence 1.5 times worse. However, such differences do not exist in the case of Phagophore, 0.12 (0.07 std.) for CDL and 0.13 (0.065 std.) for CIP. On the right, detected by humans but not by the method (false negative). In the detection of Autolysosome, the ratio of CDL is 0.12 (0.03 std.) that rises to 0.294 (0.03 std.) in CIP, hence CIP has 2.33 times worse. Finally, for Phagophore detection, the ratio of CDL is 0.36 (0.14 std.) and 0.539 (0.11 std.) for CIP, making the CIP method 1.4 times worse. In Fig. 3C, the total number of events that overlap with human-made BB is presented. The overlap percentage is divided into two ranges: 0.1 to 0.49 (lower), and 0.5 to 1 (higher). For both classes and overlapping ranges, the number of detected events is higher in the CDL segmentation than in the CIP one. The number of detected Phagophore events is in general higher than in the Autolysosome event, as expected due to its higher frequency for Autolysosome events^13^. This tendency remains when the data is normalised using the total number of events. It yielded 64.48% of detection overlap between CIP and manual detection, in particular, 62.96% in Autolysosome detection and 66.79% in Phagophore. The detection overlap of autophagy events between CDL prediction and the manually curated dataset was 77.19%. This corresponds to 78.84% for Autolysosome and 75.55% for Phagophore. As a result, the CDL method increased by 12.71% in total; 15.88% for Autolysosome and 8.76% for Phagophore with respect to the data used for its training.

#### Quantitative analysis: Overlapping quantification using manual correction

Finally, both methods were compared at pixel level using the Sørensen-Dice coefficient^56^ employing 306 manual corrected masks as previously described.The table D in Fig. 3 presents the general metrics results of both methods. The CDL method scored higher than CIP for all the metrics, the smaller differences were observed with the least recommended aggregations (the Global Accuracy and the Weighted IoU, with an increase of 1.7% and 3.05% respectively), while when using metrics that properly handle class imbalance scenarios the differences raised to 12% (12%, 12.2% and 12.19% for Mean Accuracy, Mean IoU and Mean BFS respectively. This can be explained by the fact that each metric is analysed per class, and some metrics are less class sensitive leading to a reduced variation (Fig. 3E the bottom-table). Background scores are in general very high ranging from 0.989 in the CDL Mean Accuracy to 0.893 in the Mean BFScore for CIP. Hence, as expected the score variation based on the metric employed for this class are smaller 0.3% in the Accuracy, 1.6% in the IoU and 4.9% in the BFS. The biggest differences are found in the studied events, Phagophore and Autolysosome. For the Phagorore, the increment is 21.4%, 13.8% and 12.8%, and 14.2% 21.1% and 26.9% of the Autolysosome for the Mean Accuracy, Mean IoU and Mean BFS respectively.

### Part B: Semantic segmentation performance evaluation of CIP, CDL and MDL

**Figure 4:**
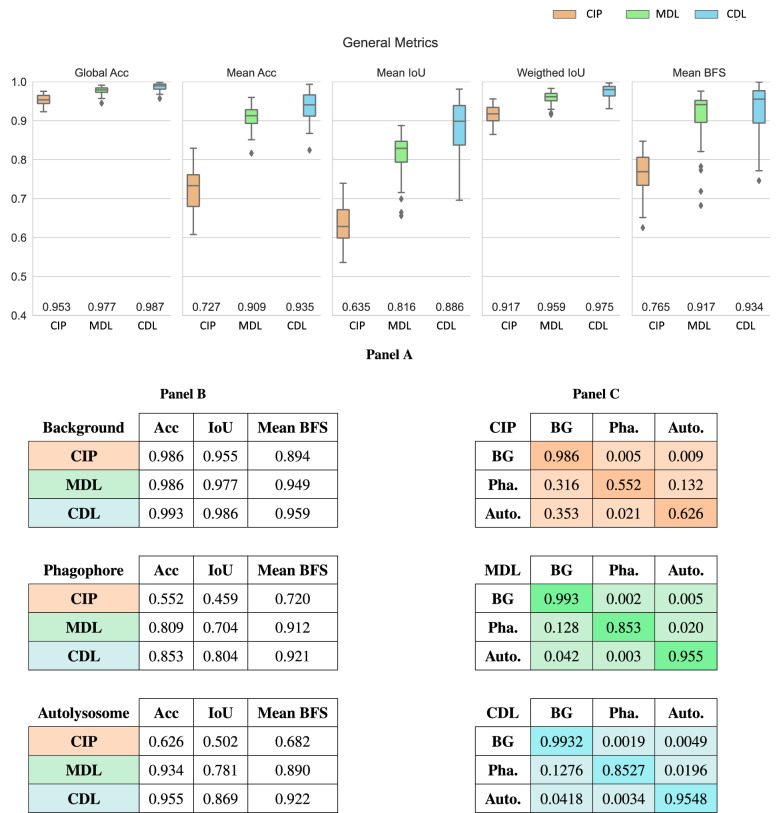
Comparison of Segmentation accuracy of CIP vs CDL vs MDL. (**A**) Box plot of the general metrics scores of the three different approaches. Mean aggregation is recommended over Global and Weighted for our strong-class imbalanced dataset. (**B**) Accuracy, Intersection over Union and Mean BFS of the three methods for each class: Background, Phagophore and Autolysosome. The large class imbalance between background and the other classes explains the small differences in performance for the background class. In these regards, Mean BFS is a more representative metric for imbalanced scenarios. (**C**) Confusion matrix for each approach. Each row of the matrix represents the instances in a predicted class while each column represents the instances in an actual class. In general, CIP scores the lowest, followed by CDL and MDL score the highest.

Once the generalisation capability of CNNs was proved in the CDL approach, the three proposed methods, the traditional CV approach (CIP), the CDL approach and a MDL trained with a small but highly curated dataset by four different experts were evaluated. The segmentation performance of the three proposed methods was assessed using 10% of the manually curated masks (31 in total) using the Sørensen-Dice coefficient^56^ (Fig. 4). In panel A, the general metrics of the different methods are presented: CIP method scored the lowest followed by MDL and CDL, being the difference of the last two almost equivalent. Taking CIP as a reference, a similar pattern that in the previous pixel-by-pixel comparison is found: the metrics that are affected by high-class imbalance (the Global accuracy and the Weighted IoU) present the smaller improvements (2.38% for DL and 3.33% for CDL; 4.21% for DL 5.82% for CDL for Global accuracy and the Weighted IoU respectively). In contrast, higher variations are found in the mean accuracy: 18.81% for MDL and 21.23% for CDL. Similarly, 18.18% for DL and 24.75% for CDL in the mean IoU. And in the same line, 15.09% for MDL and 16.86% for CDL in the mean BFScore. As mention before, the more recommended metrics in a strong class imbalance scenario such as the employed dataset, BFScore is the more recommended metric.

The aforementioned variations in the score of the first group of metrics (Global accuracy and the Weighted IoU) and the second group (mean Accuracy, IoU and BFScore) can be explained with the broken-down metrics per-class presented in Fig. 4B. Briefly, the segmentation produced with the MDL method respect to CIP presents lower variations in Background (a maximum variation of 5.48% in metric Mean BFS), and increased variations with the studied events: for the Phagophore event (maximum variation of 30.77 % in Accuracy) and Autolysosome (maximum variation. of 25.66% in Accuracy). Similarly, the CDL approach provides better general performance: With maximum variation of 6.47% for Background in Mean BFS metric, that increases to 36.6% in IoU metric for the Phagophore class and 34.5% for the IoU metric in the Autolysosome event.

Finally, in Fig. 4C, the confusion matrix of each approach is depicted. Each row of the matrix represents the instances in a predicted class while each column represents the instances in an actual class. An improvement for both Phagophore and Autolysosome classes can be observed. In particular, when CIP fails to classify these two classes, they are mostly wrongly classified as background. CIP true positives for these two classes range 0.55 to 0.6, respectively. Conversely, MDL and CDL decreased this type of error, providing 0.85 and 0.95 true positives for the same classes, respectively. We speculate that the bigger size of Autolysosome events explains the higher accuracy for this class.

### Performance comparison

Time comparison is reported for the dataset preparation stage, algorithm development and inference time. The time for the dataset preparation depends on the method. While in the case of the CIP solution, no data set preparation is needed, the CDL dataset (based on CIP), can range from no-preparation if the CIP solution is already available, to the full CIP development time (around 3 months). The MDL is one of the most common approaches when developing a DL solution, but also the most time-consuming and costly since it is conducted by experts in the biomedical field. Solution development for each method has several factors that influence the time needed. For the CIP solution, the developer’s expertise, task difficulty and final accuracy play a major role. For CDL, during training with noisy data, the training evaluation must be considered as an important factor that can increment the time and difficulty of the training. And for MDL, regular time that takes for DL optimisations. Regarding inference’s time, traditional CV, such as CIP, generalisation has short inference times but long pipelines such as the one employed in this study can last longer. The CIP solution lasted approximately 40 minutes on a conventional computer. Solutions based on DL, such as CDL and MDL, have an inference’s time of few seconds.

The human resources required in the different tasks ranged from seconds to several minutes per quarter image: The segmentation evaluation by experts took from 30 seconds to 1 minute; the generation of the Bounding Boxes around the study events from 3 to 6 minutes; manual correction per quarter images take 5 to 10 minutes; and manual curation (from Scratch) takes 20 to 40 minutes for segmentation per quarter image. Comparison of the computational resources is as follow: For computational resources, its should be considered the availability of special computer resources. such as GPUs for the training of the DL-based solutions. While inference of DL-based solutions can be done on a normal computer they can be speed up using GPUs. In a similar manner, GPUs can be also employed to speed up CIP solutions to save time if there are tasks that can be parallelized. Translating optimal settings into terms of prediction times, the traditional CV (CIP) takes 15 to 20 minutes per prediction, while DL-based systems show a significant reduction in time (CDL and MDL are 2 to 5 seconds per image).

## Discussion

High-throughput screening (HTS) is a cutting-edge technology that integrates robotics, microscopy and image analysis currently employed to study complex systems. One of the crucial steps to produce a high-quality analytical procedure relies on the development of an accurate, automatic and user-friendly system to analyse high-content images^59^. The analysis of complex high-throughput high-content microscopy images has been automatically analysed using digital image processing, highlighting the use of DL in the last years due to its superiority in results. However, during DL development and application to real datasets, several problems arise, such as the high cost of biomedical datasets^3^, noisy label data, incorrect segmentation^2^, and the lack of an easy to use platform for implementing in experimental laboratories. Hence, in this work, we explored different solutions for each challenge suiting different scenarios and packed them into an easy-to-use tool.

We have first shown that CDL can overcome some of the inaccuracies of noisy labelled mask datasets produced with conventional image processing techniques in complex images such as fluorescent microscopy images (Fig. 3A–E). Such capacity was measured using three different techniques, having each technique its advantages and disadvantages. The expert rating offers a qualitative/semi-quantitative way of evaluating the results, as illustrated in Fig. 3A. This evaluation approach is the fastest and easiest strategy for objectively measuring the difference between the methods, making use of the more intuitive human ability to spot errors and variations. All four experts confirmed in the double-blind test that there was a significant improvement in the generalisation done by the CDL compared to the CIP.

In the second approach, we make use of the fact that detection accuracy is a surrogate measure of the segmentation. BB generation is way faster than pixel-by-pixel segmentation, allowing for evaluating more images than by manual segmentation comparison.Additionally, once BBs masks are generated for a particular set of images, this can be used to compare a limitless number of methods or network states. Using this method, two important aspects where evaluated, in first place the number of BBs detected by the two methods at comparison (CIP vs CDL) but not by humans (gold standard) and vice versa, detected by the method but not by humans. This results shown superiority of the CDL method respect to CIP respect to false positive and false negative using the BBs are a subrogated metric, as depited in Fig. 3B. In second place, the degree of overlapping of the BBs, that shown a more moderated capacity for both degrees of covering (lower and higher) and for both events (Phagophore and Autolysosome), Fig. 3C.

Lastly, we evaluated the segmentation quality pixel-by-pixel label using the Sørensen-Dice coefficient (Fig. 3D,E). This method is the most common and accurate approach. However, in the context of noisy label data, the generation of good references is a limitation due to its cost and time required. Time and effort decrease considerably by producing high-quality image segmentation using deep learning techniques to assist the production of the datasets. This is in line with the improvement in the different metrics of the CNN generalisation. As expected the increase in the accuracy, was higher in the less frequent and hence more complicated classes, Phagophore and Autolysosome, which improved in a similar way. While evaluating this, it is important to remember that the most recommended metrics in a class imbalance scenario, such as the employed datasets, are one that present the highest variation. Our results present a great improvement from CIP with respect to the two methods that employ DL, while the generation of the employed dataset for training in CDL and MDL differ, the final results present similar scores in the metrics.

An important aspect that needs to be considered when working with weak labels is to find the right balance between a good generalisation of the semantic segmentation and learning the incorrect parts of the weakly labelled dataset employed, for both training and testing. This means a perfect fit of the network to the (weak) training data would yield a sub-optimal result, as it would learn to reproduce all the mistakes of the weak training data instead of learning to generalised from them. These phenomena were described as *trusting the teacher too much* in the literature^60^. Interestingly, we observed that using *less* training samples was helpful towards this end, which is in contrast to the normal overfitting problem, where in general *more* training data tends to reduce overfitting. Further studies are needed to determine the right size of the training samples.

After the CDL approach improved the CNN capacity to overcome errors, we compared which approach (CIP, MDL or CDL) is better, not only in terms of accuracy but also time and resources. Using the Sørensen-Dice coefficient for a pixel-by-pixel evaluation CIP scored the lowest, CDL the highest and MDL in between. It should be noticed that the evaluation dataset is 10% of the previous data set since the rest was employed to train the MDL solution. Such difference in the dataset size explains the variations between the Figs. 3C,D and 4. Additionally, to make a fair comparison between the two networks (MDL and CDL) some important points need to be considered. Despite having the same architecture (U-net with class-sensitive loss function) and similar training conditions (adapted to the dataset size in each case) there were major differences in the datasets used for training. Such variations refer mainly to the dataset size and the consistency of the labels. In terms of data size, the CDL approach was trained using 4000 HTS images of 680 x 512 while the MDL approach employs 274 images of 340 × 256. Regarding label consistency, although MDL employs a manually corrected dataset expecting to contain fewer errors, the consistency of the human-curated labels is lower than the labels generated with the CIP algorithm. The differences in these two datasets draw a clear line between two common scenarios of real-world experimental laboratories. Human curated data is the gold standard for ML datasets including expert knowledge label generation across domains such as medicine or biology. Notwithstanding, recent works show that automatic analysis of images using CV can excel human perception in cellular image analysis^61^, CNN can surpass human performance on visual recognition tasks^62^, and can even recognise cell structures that trained humans cannot spot^63^. Additionally, high inter-reader variability is reported in biomedical image segmentation^11^. This is especially challenging when the contours are not well defined due to the ambiguity to set the limits by the experts^12^, being the lack of label consistency a major factor that reduces algorithm performance^64^^65^. Despite DL can out-perform human tasks, especially at the pixel level, at the current state of AI development, human knowledge still needs to be included in the loop^31^. In the CDL approach, human-in-the-loop is introduced for the tasks better suited for humans, such as error spotting and critical evaluation of the predictions^66^, leaving the parts where CV techniques (traditional and AI-based) the parts were computer image processing shines at. To sum up, the CIP approach based on traditional CV techniques is the less accurate solution, requiring as well more execution time to produce the output.

It’s also important to consider the time needed to develop the CIP approach. Estimation of this is hard to assess since its highly dependent on the context which in our case was the initial time was 3 months. This could be less if there was a pipeline already in place that can be fine-tuned.

However, no training set is needed, neither are special computational resources such as dedicated Graphics Processing Units (GPUs). The MDL approach employs a small but manually curated dataset that offers a more accurate solution and faster prediction times. In this case, the generation of manual labels is very costly and often contains label inconsistencies. Additionally, it requires specific computational resources such as GPUs for model training. Finally, the CDL approach is the most balanced solution. It is as fast as the MDL approach, requires the same resources, but circumvents the issues concerning human-generated labels. Each laboratory setting comes with compromises. Such considerations should be weighed to justify choosing one approach over the other depending on the setting needs. All in all, the CDL approach offers the best trade-off.

## Conclusion

In this work, we have developed a tool that outperforms the previous solution (CIP) in three different aspects: accuracy, speed and usability. We have reached better segmentation performance starting with noisy label data generated with CIP, which was leveraged by CNN capacities, overcoming the errors and generalising beyond the provided noisy labels. We discuss the pros and cons of traditional CV-based and DL-based solutions and combine the best of both methodologies in the CDL approach. The shortage of gold standard datasets is one of the main concerns when training solutions with noisy label data. In this work, we implemented three independent but complementary methods with different advantages and disadvantages. We also addressed another big obstacle that limits the usage of DL solutions in experimental laboratories. Such solutions often require special IT skills for their deployment and use. In this sense, we embedded our solution into a user-friendly GUI tool for MATLAB. Finally, we anticipate that these results might be generalised to other domains other than HTS imaging. This work aims to close the gap between new technologies and their implementation in real scenarios in HTS microscopy image analysis.

## Future work and Limitations

This paper reports a case study of our experience developing a solution for a real laboratory, and focus on how to obtain the best results with limited resources, something that we believe is under represented in many research papers. We believe these insights can be highly valuable for people in similar scenarios. However, the conclusions should be taken from a case of study perspective, opening very interesting ideas that need to be validated with further research. Future work may include a more detailed analysis on how U-net is having such good results in generalisation over noisy label data. This includes detailed results about their generalisation such as statistical analysis on outliers, average and good results on the different approaches using synthetic data that mimic the current scenario.

## Supplementary Information

Supplementary Information 1.

## Data Availability

Code and data will be available thought the R3 platform of the University of Luxembourg. The GUI presented in this paper can be found: https://beatrizgsc.github.io/CellSegmentation/.

## References

[1] Starkuviene V, Pepperkok R (2007). The potential of high-content high-throughput microscopy in drug discovery. Br. J. Pharmacol..

[2] Karimi D, Dou H, Warfield SK, Gholipour A (2020). Deep learning with noisy labels: Exploring techniques and remedies in medical image analysis. Med. Image Anal..

[3] Koohbanani NA, Jahanifar M, Tajadin NZ, Rajpoot N (2020). Nuclick: A deep learning framework for interactive segmentation of microscopic images. Med. Image Anal..

[4] Rosenfeld A (1969). Picture processing by computer. ACM Comput. Surv..

[5] Krizhevsky A, Sutskever I, Hinton GE (2012). Imagenet classification with deep convolutional neural networks. Adv. Neural Inf. Process. Syst..

[6] OMahony, N. *et al.* Deep learning vs. traditional computer vision. In *Science and Information Conference* 128–144 (Springer, 2019).

[7] Wienert S (2012). Detection and segmentation of cell nuclei in virtual microscopy images: A minimum-model approach. Sci. Rep..

[8] Lempitsky, V., Vedaldi, A. & Zisserman, A. Pylon model for semantic segmentation. In *Advances in Neural Information Processing Systems* 1485–1493 (2011).

[9] LeCun Y, Bengio Y, Hinton G (2015). Deep learning. Nature.

[10] Xing F, Xie Y, Su H, Liu F, Yang L (2017). Deep learning in microscopy image analysis: A survey. IEEE Trans. Neural Netw. Learn. Syst..

[11] Menze BH (2014). The multimodal brain tumor image segmentation benchmark (brats). IEEE Trans. Med. Imaging.

[12] Kats, E., Goldberger, J. & Greenspan, H. A soft staple algorithm combined with anatomical knowledge. In *International Conference on Medical Image Computing and Computer-Assisted Intervention* 510–517 (Springer, 2019).

[13] Arias-Fuenzalida J (2019). Automated high-throughput high-content autophagy and mitophagy analysis platform. Sci. Rep..

[14] Torrey, L. & Shavlik, J. Transfer learning. In *Handbook of Research on Machine Learning Applications and Trends: Algorithms, Methods, and Techniques* 242–264 (IGI global, 2010).

[15] Shorten C, Khoshgoftaar TM (2019). A survey on image data augmentation for deep learning. J. Big Data.

[16] Jiang, L., Zhou, Z., Leung, T., Li, L.-J. & Fei-Fei, L. Mentornet: Learning data-driven curriculum for very deep neural networks on corrupted labels. In *International Conference on Machine Learning* 2304–2313 (PMLR, 2018).

[17] Zhou Z-H, Liu X-Y (2005). Training cost-sensitive neural networks with methods addressing the class imbalance problem. IEEE Trans. Knowl. Data Eng..

[18] Yu S (2020). Robustness study of noisy annotation in deep learning based medical image segmentation. Phys. Med. Biol..

[19] Freytag, A., Rodner, E. & Denzler, J. Selecting influential examples: Active learning with expected model output changes. In *European Conference on Computer Vision* 562–577 (Springer, 2014).

[20] Thrun, S., Saul, L. K. & Schölkopf, B. *Advances in Neural Information Processing Systems 16: Proceedings of the 2003 Conference*, Vol. 16 (MIT press, 2004).

[21] Käding, C. *et al.* Active learning for regression tasks with expected model output changes. In *BMVC* 103 (2018).

[22] Pop, R. & Fulop, P. Deep ensemble bayesian active learning: Addressing the mode collapse issue in monte carlo dropout via ensembles. *arXiv preprint* arXiv:1811.03897 (2018).

[23] Parisi GI, Kemker R, Part JL, Kanan C, Wermter S (2019). Continual lifelong learning with neural networks: A review. Neural Netw..

[24] Pianykh OS (2020). Continuous learning AI in radiology: Implementation principles and early applications. Radiology.

[25] Bengio, Y., Louradour, J., Collobert, R. & Weston, J. Curriculum learning. In *Proceedings of the 26th Annual International Conference on Machine Learning* 41–48 (2009).

[26] Tang P, Yan X, Liang Q, Zhang D (2021). Afln-dgcl: Adaptive feature learning network with difficulty-guided curriculum learning for skin lesion segmentation. Appl. Soft Comput..

[27] Wei, J. *et al.* Learn like a pathologist: curriculum learning by annotator agreement for histopathology image classification. In *Proceedings of the IEEE/CVF Winter Conference on Applications of Computer Vision* 2473–2483 (2021).

[28] Wang L, Yoon K-J (2021). Knowledge distillation and student-teacher learning for visual intelligence: A review and new outlooks. IEEE Trans. Pattern Anal. Mach. Intell..

[29] Li, Y. *et al.* Learning from noisy labels with distillation. In *Proceedings of the IEEE International Conference on Computer Vision* 1910–1918 (2017).

[30] Arani, E., Sarfraz, F. & Zonooz, B. Improving generalization and robustness with noisy collaboration in knowledge distillation. *arXiv preprint* arXiv:1910.050571 (2019).

[31] Holzinger A (2016). Interactive machine learning for health informatics: When do we need the human-in-the-loop?. Brain Inform..

[32] Song H, Kim M, Park D, Shin Y, Lee J-G (2022). Learning from noisy labels with deep neural networks: A survey. IEEE Trans. Neural Netw. Learn. Syst..

[33] Lynch-Day MA, Mao K, Wang K, Zhao M, Klionsky DJ (2012). The role of autophagy in Parkinsons disease. Cold Spring Harbor Perspect. Med..

[34] Rosado C, Mijaljica D, Hatzinisiriou I, Prescott M, Devenish RJ (2008). Rosella: A fluorescent ph-biosensor for reporting vacuolar turnover of cytosol and organelles in yeast. Autophagy.

[35] Cheng, P.-C. The contrast formation in optical microscopy. In *Handbook of Biological Confocal Microscopy* 162–206 (Springer, 2006).

[36] Gonzalez, R. C. & Woods, R. E. Thresholding. *Digital Image Processing* 595–611 (2002).

[37] Haddad RA, Akansu AN (1991). A class of fast gaussian binomial filters for speech and image processing. IEEE Trans. Signal Process..

[38] Soille, P. *Morphological Image Analysis: Principles and Applications* (Springer Science & Business Media, 2013).

[39] Gonzalez RC, Woods RE, Eddins S (2002). Image segmentation. Dig. Image Process..

[40] Davidson, M. W. & Abramowitz, M. *Molecular Expressions Microscopy Primer: Digital Image Processing-difference of Gaussians Edge Enhancement Algorithm* (Olympus America Inc., and Florida State University, 2006).

[41] Dogra A, Bhalla P (2014). Image sharpening by gaussian and Butterworth high pass filter. Biomed. Pharmacol. J..

[42] Chen X, Yang J, Wu Q, Zhao J, He X (2012). Directional high-pass filter for blurry image analysis. Signal Process..

[43] Long, J., Shelhamer, E. & Darrell, T. Fully convolutional networks for semantic segmentation. In *Proceedings of the IEEE Conference on Computer Vision and Pattern Recognition* 3431–3440 (2015). 10.1109/TPAMI.2016.2572683 27244717

[44] Ronneberger, O., Fischer, P. & Brox, T. U-net: Convolutional networks for biomedical image segmentation. In *International Conference on Medical Image Computing and Computer-assisted Intervention* 234–241 (Springer, 2015).

[45] Falk T (2019). U-net: Deep learning for cell counting, detection, and morphometry. Nat. Methods.

[46] Jadon, S. A survey of loss functions for semantic segmentation. In *2020 IEEE Conference on Computational Intelligence in Bioinformatics and Computational Biology (CIBCB)* 1–7 (IEEE, 2020).

[47] Ling, C. X. & Sheng, V. S. Cost-sensitive learning. *Encyclopedia of Machine Learning* 231–235 (2010).

[48] Isensee, F., Jaeger, P. F., Kohl, S. A., Petersen, J. & Maier-Hein, K. H. nnu-net: a self-configuring method for deep learning-based biomedical image segmentation. *Nat. Methods* 1–9 (2020). 10.1038/s41592-020-01008-z 33288961

[49] Onofrey, J. A. *et al.* Generalizable multi-site training and testing of deep neural networks using image normalization. In *2019 IEEE 16th International Symposium on Biomedical Imaging (ISBI 2019)* 348–351 (IEEE, 2019). 10.1109/isbi.2019.8759295 PMC7457546 32874427

[50] Perez, L. & Wang, J. The effectiveness of data augmentation in image classification using deep learning. *arXiv preprint* arXiv:1712.04621 (2017).

[51] Sutskever, I., Martens, J., Dahl, G. & Hinton, G. On the importance of initialization and momentum in deep learning. In *International Conference on Machine Learning* 1139–1147 (2013).

[52] Krogh A, Hertz J (1991). A simple weight decay can improve generalization. Adv. Neural Inf. Process. Syst..

[53] Agrawal, P., Shriwastava, S. & Limaye, S. Matlab implementation of image segmentation algorithms. In *2010 3rd International Conference on Computer Science and Information Technology*, Vol. 3, 427–431 (IEEE, 2010).

[54] Massey FJ (1951). The Kolmogorov–Smirnov test for goodness of fit. J. Am. Stat. Assoc..

[55] Arteta, C. *Computer Vision and Machine Learning for Microscopy Image Analysis*. Ph.D. thesis, University of Oxford (2015).

[56] Sorensen, T. “a method of establishing groups of equal amplitude in plant sociology based on similarity of species and its application to analyses of the vegetation on danish commons”. *Det Kongelige danske videnskabernes selskab* (1948).

[57] Csurka, G., Larlus, D., Perronnin, F. & Meylan, F. What is a good evaluation measure for semantic segmentation?. In *Proceedings of the British Machine Vision Conference* (BMVA Press, 2013).

[58] Reinke, A. *et al.* Common limitations of image processing metrics: A picture story. *arXiv preprint* arXiv:2104.05642 (2021).

[59] Pereira D, Williams J (2007). Origin and evolution of high throughput screening. Br. J. Pharmacol..

[60] Fedorov, A. *et al.* End-to-end learning of brain tissue segmentation from imperfect labeling. In *2017 International Joint Conference on Neural Networks (IJCNN)* 3785–3792 (IEEE, 2017).

[61] Danuser G (2011). Computer vision in cell biology. Cell.

[62] Lundervold AS, Lundervold A (2019). An overview of deep learning in medical imaging focusing on MRI. Z. Med. Phys..

[63] Dance A (2021). Ai spots cell structures that humans can’t. Nature.

[64] Zhang, L. *et al.* Disentangling human error from the ground truth in segmentation of medical images. *arXiv preprint* arXiv:2007.15963 (2020).

[65] Sommer C, Gerlich DW (2013). Machine learning in cell biology-teaching computers to recognize phenotypes. J. Cell Sci..

[66] Gurari D, Zhao Y, Jain SD, Betke M, Grauman K (2019). Predicting how to distribute work between algorithms and humans to segment an image batch. Int. J. Comput. Vis..

