# Supplementary material for: Generalising from conventional pipelines using deep learning in high-throughput screening workflows
Source: Sci Rep. 2022 Jul 6;12:11465. doi: 10.1038/s41598-022-15623-7 (PMC9259641; doi:10.1038/s41598-022-15623-7)
Supplement: Supplementary file 1 — Supplementary Information 1. [file 41598_2022_15623_MOESM1_ESM.pdf]

SUPPLEMENTARY MATERIAL

Generalising from conventional pipelines using deep learning in high-throughput screening workflows

Beatriz Garcia-Santa Cruz [1,2,✉], Jan Sölter [2], Gemma Gomez Giro [3], Claudia Saraiva [3], Sonia Sabate-Soler [3], Jennifer Modamio [3], Kyriaki Barmpa [3], Jens Christian Schwamborn [3], Frank Hertel [1,2], Javier Jarazo [3,4,*], Andreas Husch [2,5,*,✉]

[1] National Department of Neurosurgery, Centre Hospitalier de Luxembourg, 4, Rue Ernest Barble, L-1210 Luxembourg (City), Luxembourg

[2] Interventional Neuroscience Group, Luxembourg Center for Systems Biomedicine, University of Luxembourg, 6, Avenue du Swing, L-4367 Belvaux, Luxembourg

[3] Developmental and Cellular Biology, Luxembourg Center for Systems Biomedicine, University of Luxembourg, 6, Avenue du Swing, L-4367 Belvaux, Luxembourg

[4] OrganoTherapeutics SARL, 6A, avenue des Hauts-Fourneaux, L-4365 Esch-sur-Alzette, Luxembourg

[5] Systems Control Group, Luxembourg Centere for Systems Biomedicine, University of Luxembourg, 6, Avenue du Swing, L-4367 Belvaux, Luxembourg

[*] Contributed equally

Corresponding Authors (✉) garciasantacruz.beatriz@gmail.com or andreas.husch@uni.lu

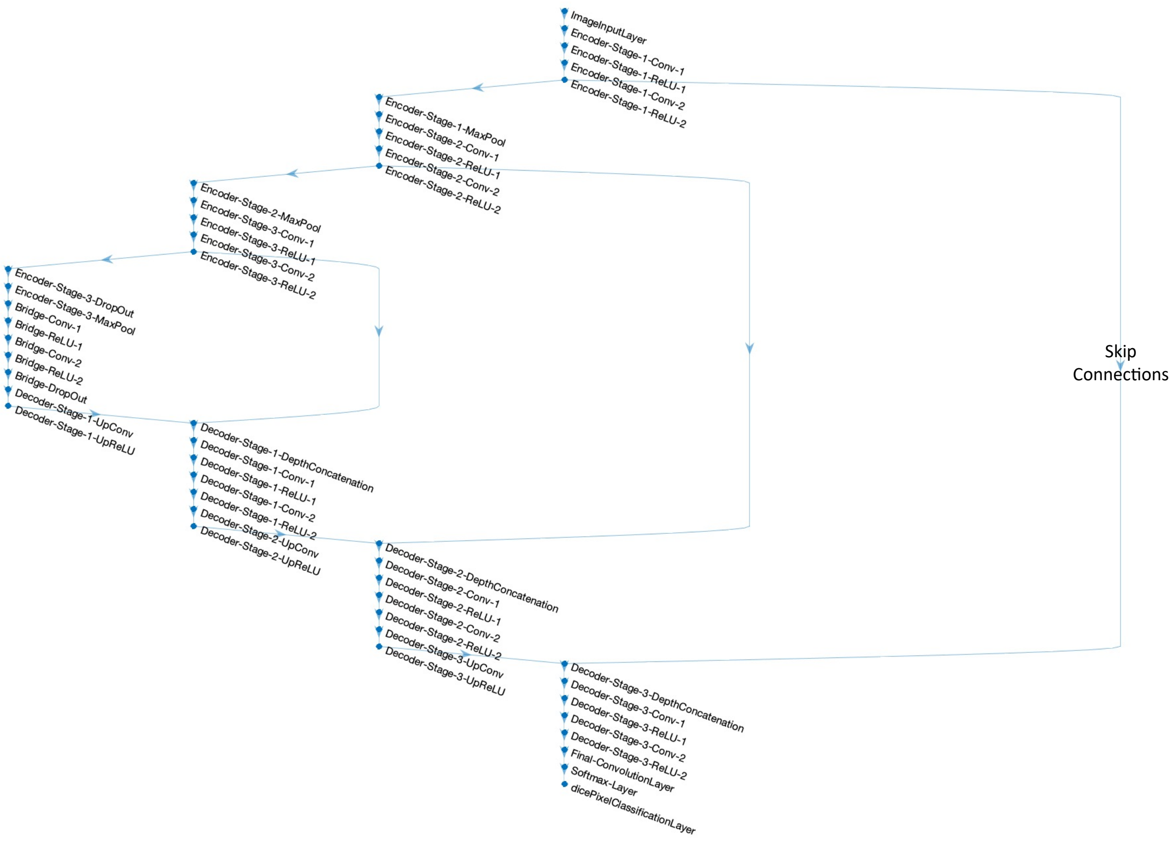

Supplementary Figure S1: Graphical representation of the employed convolutional neural network. Modified U-net, 3 level encoder-decoder with skip connections. Our modification consisted of the final dice Pixel Classification layer to increase the accuracy in the high-class imbalance scenario of our dataset.

2.a

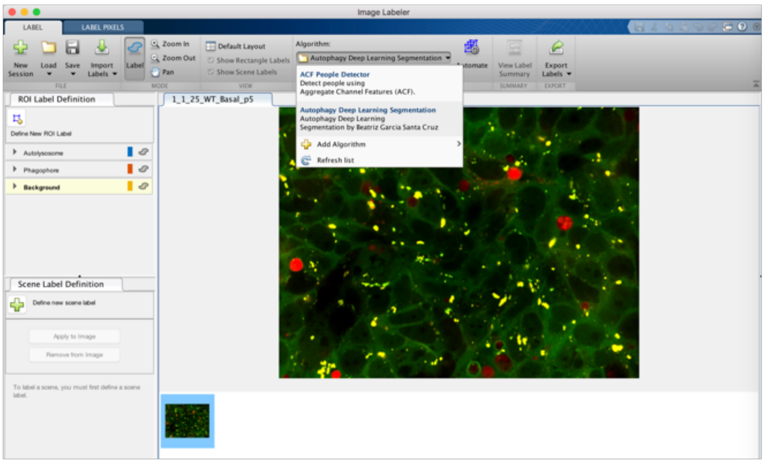

2.b

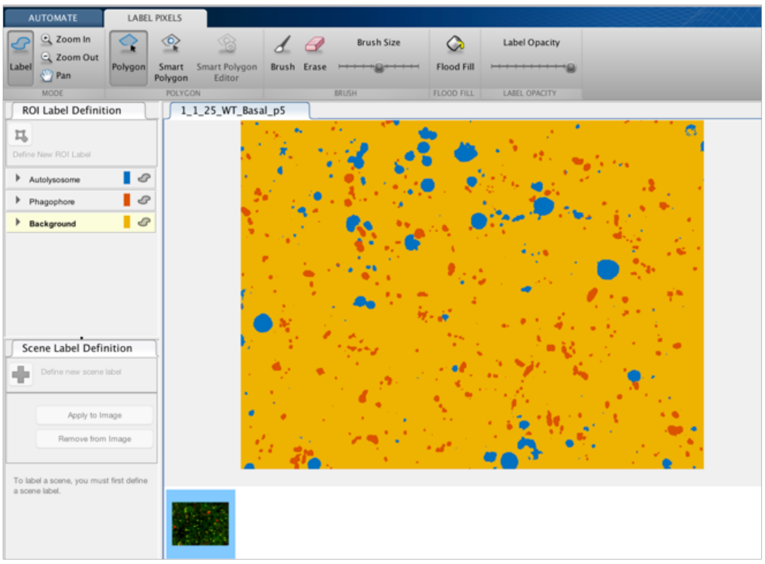

2.c

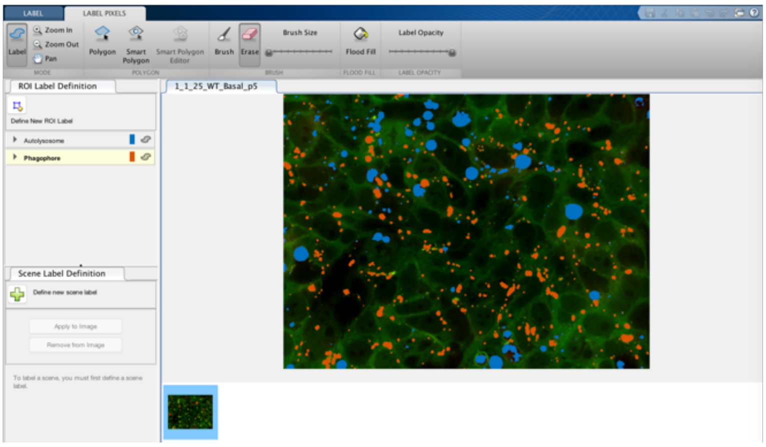

Supplementary Figure S2: Different stages of the User-friendly GUI developed in MATLAB. 2a: After loading the image and define the labels, select the segmentation algorithm. 2b: Example of the generated segmentation mask within few seconds. 2c: Example of one class removal (Background in the case), the tools on the top such as bush tools, lasso tools, superpixel for facile correction.

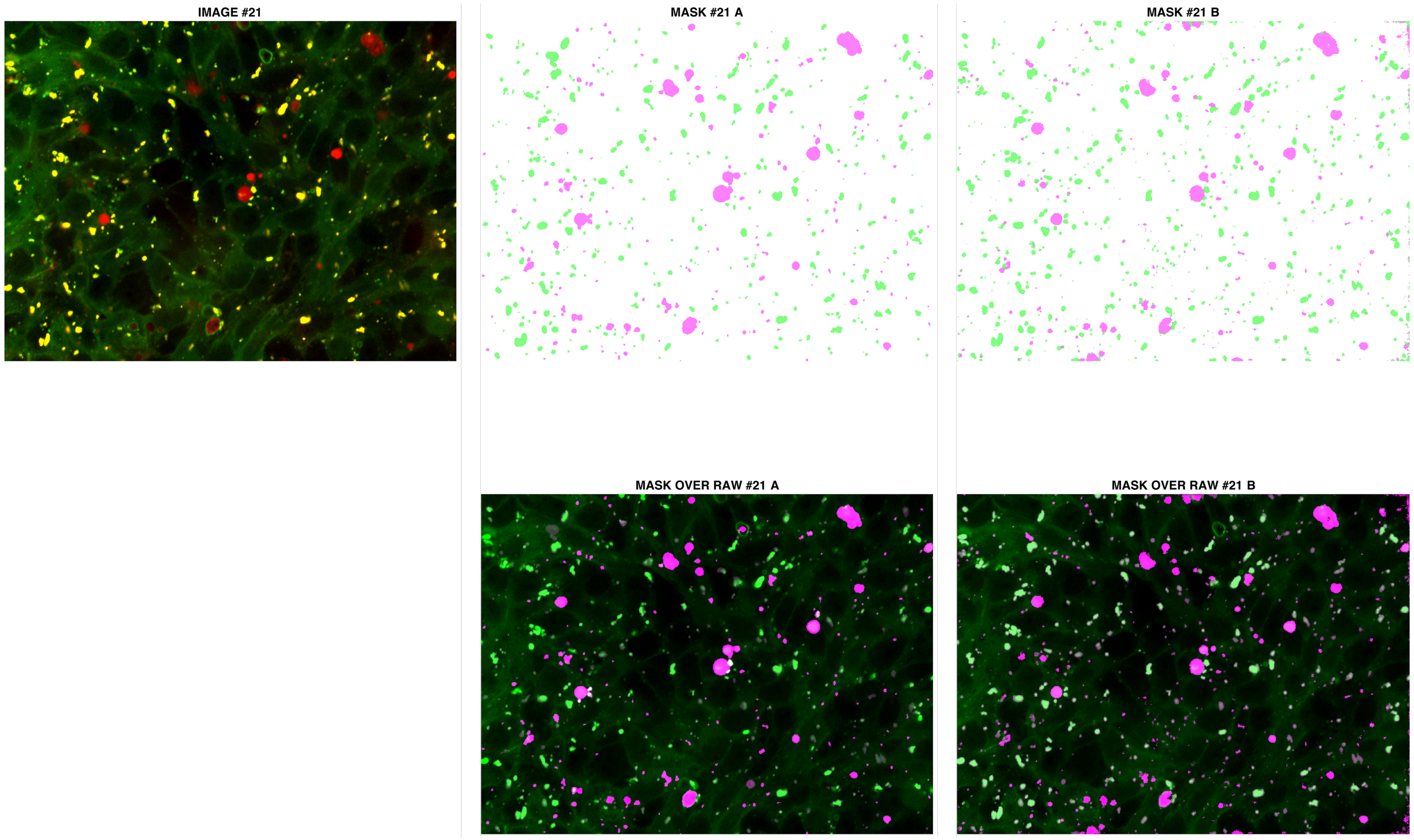

Supplementary Figure S3: Graphical example of one sample used during quantitative analysis given to the experts. On the left: Original raw image. On the middle and the right, the segmentations to rate. Top: predicted segmentation (Phagophore and Autolysosome), bottom the overlay with the raw image. The order of the method employed to general masks A and B was double blinded.
